# Major Factors Contributing to Positive and Negative Childbirth Experiences in Pregnant Women Living with HIV

**DOI:** 10.3390/bs15040442

**Published:** 2025-03-31

**Authors:** Andréa Paula de Azevedo, Luisa Castro, Cristina Barroso Hofer, Francisca Rego

**Affiliations:** 1 Faculty of Medicine, University of Porto, 4099-002 Porto, Portugal; luisacastro@med.up.pt (L.C.); mfrego@med.up.pt (F.R.); 2 Martagão Gesteira Childhood and Pediatric Institute, Federal University of Rio de Janeiro, Rio de Janeiro 21941-853, Brazil; cbhofer@hucff.ufrj.br; 3 CINTESIS@ RISE, Faculty of Medicine, University of Porto, 4099-002 Porto, Portugal; 4 Faculty of Medicine, Federal University of Rio de Janeiro, Rio de Janeiro 21941-853, Brazil

**Keywords:** childbirth, HIV, obstetrics, patient satisfaction, positive experience, negative experience

## Abstract

Objective: The aim of this study was to assess the opinions of pregnant women living with HIV (PWLWHIV) about their positive childbirth experiences and the most important factors contributing to positive or negative experiences. Methods: A cross-sectional study was conducted with 82 PWLWHIV; semi-structured interviews were conducted in a public hospital in Rio de Janeiro. Results: A total of 65 (79.3%) PWLWHIV experienced a positive childbirth experience. Conversely, 14 (17.1%) PWLWHIV had a negative experience. The main reasons given by the PWLWHIV for positive experiences were the good health of the baby, their partner’s presence at the childbirth, and good healthcare professional support. The main reasons for negative childbirth experiences were poor healthcare professional support, excessive pain or medication, and the absence of a companion during childbirth. Conclusions: Our findings indicate that the health of the baby at birth was the main factor in positive childbirth experiences. On the other hand, poor healthcare professional support was the main cause of negative childbirth experiences. Increasing the incidence of positive childbirth experiences could reduce maternal depression and anxiety, and significantly impact neonatal outcomes (mainly low birth weights and preterm birth). Future studies should target reducing depressive symptoms in perinatal HIV-positive women, increasing partner involvement, and decreasing HIV stigma.

## 1. Objective

The aim of this study was to determine the most important factors for positive and negative childbirth experiences in pregnant women living with HIV (PWLWHIV).

## 2. Introduction

The incidence of HIV among pregnant women is influenced by factors such as geographic location, access to healthcare, and sociodemographic characteristics. These findings underscore the importance of targeted HIV prevention and treatment strategies during pregnancy to reduce mother-to-child transmission and improve maternal health outcomes. A systematic review and meta-analysis reported an HIV incidence rate of 3.6 per 100 person-years among pregnant and breastfeeding women in sub-Saharan Africa, with variability between different populations (Graybill et al., 2020).

Experiences of pregnancy and birth are important and have long-term impacts on the well-being of women and their families. Perinatal services should aim for care that promotes a positive childbearing experience, as well as optimizing health outcomes for the woman and newborn (Hall et al., 2023; Leinweber et al., 2023). A positive childbirth experience is influenced by several key factors, which can be broadly categorized into healthcare provider attributes, health system attributes, communication and decision making, and the overall experience of care. These aspects encompass both the emotional and physical aspects of care (Leinweber et al., 2023). A positive childbirth experience is characterized by a combination of emotional support, respectful and competent care, effective communication, and a safe and supportive environment. These factors collectively contribute to a woman’s sense of control, safety, and respect during childbirth, leading to a positive experience (Hall et al., 2023; Hemalatha, 2012; Montgomery, 2003; Powell et al., 2017).

Competent and professional healthcare providers who offer respectful and individualized care are crucial. Providers who facilitate shared decision making and provide continuous support during labor significantly enhance the childbirth experience (Hall et al., 2023; Taheri et al., 2018). Effective communication and the involvement of women in decision-making processes are essential. Pregnant women who feel informed and involved in their care are more likely to report positive childbirth experiences (Baranowska et al., 2020; Hall et al., 2023; Nicoloro-SantaBarbara et al., 2017). Effective communication can alleviate maternal anxiety, which is often heightened in older mothers due to the increased risk of complications. Maternal age influences childbirth outcomes through increased medical risks and the need for specialized, integrated care. Effective communication and a supportive care environment are essential to mitigate these risks and enhance the childbirth experience for older mothers (Vandekerckhove et al., 2021).

A study by Nicoloro-SantaBarbara et al. demonstrated that better communication, collaboration, and empowerment from healthcare providers were associated with more frequent salutary health behavior practices in late pregnancy, mediated by reductions in anxiety (Nicoloro-SantaBarbara et al., 2017). Effective patient–provider communication is essential for older mothers, as it can reduce anxiety, set realistic expectations, and enhance understanding and decision making, thereby improving childbirth outcomes. Moreover, patient-centered communication that includes messages of empowerment, emotional support, and clear explanations can help set realistic and flexible expectations for the birthing experience (Kaimal & Norton, 2021).

The physical and psychological environment of the birth setting plays a significant role in pregnant women’s decision-making process (Chen et al., 2023). Elements such as a “homey” atmosphere, comfort, demedicalization of the birthing environment, and the presence of birth partners can improve outcomes and satisfaction. This description from mothers highlights the importance of provider interactions for facilitating a positive childbirth experience. Feeling supported and having a sense of control, safety, and respect are central tenets (Chen et al., 2023; Downe et al., 2018; Leinweber et al., 2023). The most effective strategies to create a positive birth experience are supporting women during birth, intrapartum care with minimal intervention, and birth preparedness (Taheri et al., 2018; Vandekerckhove et al., 2021).

Emotional support and addressing stigma are also vital. PWLWHIV often face significant psychological and emotional challenges, including stigma and HIV-related shame, which can impact their childbirth experience and postpartum care engagement (Fentie et al., 2022). Providing a supportive environment that includes counseling and peer support can help mitigate these challenges (Fentie et al., 2022). Maternity care should be designed to cater to or meet women’s personal and socio-cultural beliefs and expectations (Baumont et al., 2023).

Maternal stress, including stress, depression, and PTSD (post-traumatic stress disorder), from negative childbirth experiences, particularly in women living with HIV treated with antivirals, is closely associated with adverse cognitive outcomes (Boivin et al., 2019; Magondo et al., 2024; Schoeman et al., 2017). Higher maternal stress and depression scores were linked to lower overall cognitive scores in HIV-exposed infants, affecting domains such as expressive language, fine motor skills, gross motor skills, and visual reception (Mebrahtu et al., 2018; Nöthling et al., 2013). Maternal PTSD and depression are significant risk factors for child behavior problems (Nöthling et al., 2013; Rodriguez et al., 2018b). Moreover, Marr et al. found that increasing maternal stress during late pregnancy was associated with altered neonatal amygdala connectivity to the anterior insula and the ventromedial prefrontal cortex, as well as negative affect in infants, which can lead to poor long-term cognitive development (Marr et al., 2023).

High maternal HIV viremia during pregnancy can contribute to neonatal cognitive impairments, including deficits in global cognitive abilities, short-term memory, delayed memory, attention, and processing speed (Awadu et al., 2022; Benki-Nugent et al., 2017). Additionally, le Roux et al. reported that cumulative maternal viremia predicted lower motor and expressive language scores (le Roux et al., 2019). Young et al. showed that HEU children scored significantly lower on measures of Full-Scale IQ, Performance IQ, visual motor integration, and adaptive functioning during early childhood (Young et al., 2022).

In utero and peripartum exposure to certain antiretroviral therapies (ARTs) has been associated with developmental disorders. For instance, one study found that children exposed to a combination of single-dose nevirapine, zidovudine, and lamivudine (SdNVP + AZT + 3TC) had higher probabilities of attention deficit and hyperactivity disorder (ADHD), autism spectrum disorder (ASD), and functional impairment compared with those not exposed to this therapy (Awadu et al., 2022).

## 3. Material and Methods

A cross-sectional study was conducted at a public prenatal care center in Rio de Janeiro that cares for PWLWHIV (De Azevedo et al., 2025). At this institution, prenatal care is provided by specialists in obstetrics and gynecology, infectiologists, and nurses. Semi-structured interviews with the PWLWHIV were conducted during the first month of postnatal care. They were asked if they had a positive or negative experience and to give three factors that contributed to this positive or negative experience. All the interviews were conducted by one researcher, and the participants filled out the questionnaire in a quiet environment without any interruptions. The researcher remained available to answer any questions and to clarify any specific aspects of the questionnaire items. The doctors or midwives who assisted in the childbirth were not informed of this study to avoid any bias.

The inclusion criteria were PWLWHIV who were 18 years or older and had a childbirth experience less than one month ago at a public maternity ward in Rio de Janeiro.

The exclusion criteria were preterm birth and PWLWHIV who could not understand the interview and the questions.

Considering the annual number of PWLWHIV in Rio de Janeiro (N), we defined a margin of error of 4 points (resulting in a confidence interval with an amplitude of 8 points), a confidence level of 95%, and a variance of 369 (Lopes et al., 2019, 2021a, 2021b). With these parameters, the calculation indicated a minimum sample of 80 participants. Therefore, we set a minimum sample size of 80 pregnant women for this study. For this study, all PWLWHIV that met the inclusion criteria were included until the minimum size was achieved, with the enrollment period lasting from June 2023 to December 2023.

### 3.1. Instrument

A semi-structured interview was developed and conducted, which involved a questionnaire (used to collect sociodemographic data and information about the labor, the delivery, and the baby) and a question about whether the childbirth was positive, negative, or neither positive nor negative. The PWLWHIV were also asked to give the three main causes that led to their experience.

### 3.2. Data Analysis

The variables were maternal age (years); parity (primiparity or multiparity); ethnicity (white, mixed, or black); type of delivery (vaginal or cesarean); newborn sex (male or female); gestational age at birth (weeks); birth weight (grams); 1 min Apgar score (0 = score > 7; 1 = score ≤ 7); length at birth (cm); and complications during birth. Table 1 displays the variables studied: the sociodemographic and birth characteristics of the study population, which is the same as that used in a study using the Mackey questionnaire (De Azevedo et al., 2025).

When asked if their childbirth experience was positive or negative and the factors that contributed to it, the PWLWHIV were allowed to answer freely and could give one, two, or three reasons. Through content analysis, the answers were divided into 7 groups for positive experiences and 7 groups for negative experiences. Table 2 shows the answers given by the PWLWHIV and their groupings.

The factors that contributed to a positive experience were baby health; the presence of a companion; good assistance from healthcare professionals (doctors, nurses, and midwives); everything going well; good physical structure of the maternity ward; an early visit to the maternity ward during prenatal care; and a lack of pain during childbirth.

The factors that contributed to a negative experience were an absence of a companion; poor assistance from the healthcare professionals (doctors, nurses, and midwives); complications for the baby; poor physical structure of the maternity ward; excessive pain or medication during birth; an absence of information during birth; and cesarean delay.

The descriptive statistics are presented as absolute (n) and relative (%) frequencies for categorical variables, the mean and standard deviation (SD) for normally distributed quantitative variables, and the median with interquartile intervals (IQIs), minimum and maximum values, otherwise.

## 4. Results

This study was conducted on 82 PWLWHIV in public maternity wards in Rio de Janeiro. Table 1 displays the sociodemographic and birth characteristics of the study population, which is the same as that used in a study using the Mackey questionnaire (De Azevedo et al., 2025).

The participants ranged in age from 19 to 49 years, with a median age of 28.5 (IQI = [23.3; 28.6]). In terms of ethnicity, 14 were white (17.1%), 34 were mixed (41.5%), and 34 were black (41.5%). Almost half of the PWLWHIV had not completed high school (40, 48.8%) and only 2 (2.4%) completed university. The majority were married or living unofficially with a partner (58, 70.7%), and an additional 24 (29.2%) were single or divorced.

Their childbirth history ranged from one to six pregnancies, with a mean of one and a half pregnancies. Their parity ranged from zero to five. Forty-two (42, 51.2%) were pregnant with their first child. None of the women who had vaginal deliveries received either analgesia or anesthesia during the labor and birth. Only those who had a cesarean section received anesthesia (25, 30.5%).

There were no differences in the responses based on race, parity, or socio-economic status. Unfortunately, it was not possible to analyze the influence of viral load; companion support; whether it was a planned pregnancy; or smoking, alcohol, or drug use, because most of the PWLWHIV had the same profile.

**Figure 1:**
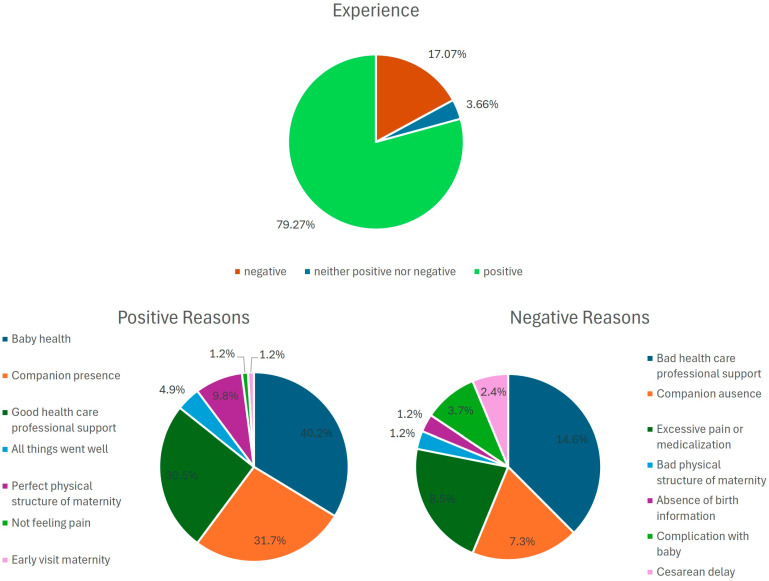
Reasons associated by PWLWHIV with positive and negative experiences in the sample (N = 82).

A positive childbirth experience occurred for 65 PWLWHIV (79.3%) (Figure 1). The main reasons given by the PWLWHIV for positive childbirth experiences were associated with the health of the baby (n = 33, 40.2%), their partner’s presence at childbirth (n = 26, 31.7%), and good support from the healthcare professionals (n = 25, 30.5%) (Figure 1). The main reasons for the negative childbirth experiences were poor healthcare professional support (n = 12, 14.6%), excessive pain or medication (n = 7, 8.5%), and the absence of a companion during childbirth (n = 6, 7.3%) (Figure 1).

Regarding whether it was the first baby or not, there was no statistical difference concerning satisfaction with childbirth. The other tables can be found in the Supplementary Material.

## 5. Discussion

For pregnant women, practical and emotional support from birth companions and competent healthcare professionals is important (Karlström et al., 2015). The presence of a supportive environment, including a trusting and respectful relationship with healthcare providers, is crucial. Additionally, effective communication and shared decision making are essential components that contribute to a positive experience (Downe et al., 2016; Hall et al., 2023).

The importance of a partner for PWLWHIV is multifaceted and significantly impacts both maternal and infant health outcomes. Male partner involvement in antenatal care has been shown to be critical in reducing the mother-to-child transmission (MTCT) of HIV and improving HIV-free survival among infants. A randomized controlled trial in South Africa demonstrated that active male partner involvement during pregnancy was associated with a significantly lower rate of HIV-infected infants at 12 months postpartum (adjusted odds ratio [aOR] = 4.55) and a lower proportion of dead and HIV-infected infants compared with those without partner involvement (aOR = 1.98) (Sifunda et al., 2019). Emotional and instrumental support from male partners is also crucial for the physical and mental health of pregnant women living with HIV. Qualitative interviews in Zambia highlighted that women prioritized communication, honesty, and respect in their relationships, and considered partner support essential for their well-being during pregnancy (Hampanda et al., 2021). Psychosocial support from partners can mitigate the stress and stigma associated with HIV during the perinatal period. In Uganda, women reported that partner support helped them navigate the challenges of HIV-related stigma and intimate partner violence, which are common stressors during pregnancy (Ashaba et al., 2017). Partner support plays a crucial role in reducing maternal stress and improving mental health for PWLWHIV. Studies have shown that male partner involvement can significantly decrease depressive symptoms in HIV-positive pregnant women.

PWLWHIV also highly valued personal achievement and control through active decision making during labor and birth, even when medical interventions were necessary. The sense of being informed and involved in decisions regarding labor and pain management significantly impacts the overall experience (Downe et al., 2016; Hall et al., 2023) (Medema-Wijnveen et al., 2012). This is in accordance with our study, as the first and third most common reasons for a negative childbirth experience were related to healthcare professionals. PWLWHIV value practical and emotional support from birth companions and competent, reassuring, and kind clinical staff. The presence of a supportive environment, including a trusting and respectful relationship with healthcare providers, is crucial. Additionally, effective communication and shared decision making are essential components that contribute to a positive experience (Kunneman & Montori, 2017; Meyer et al., 2017; Noone, 2002).

The physical environment also plays a role (fourth most common reason), with PWLWHIV emphasizing the importance of a clinically and psychologically safe environment, preserving the individuality of PWLWHIV (Watt et al., 2024).

PWLWHIV without partner support face several common psychological challenges, which can significantly impact their mental health and overall well-being. Depression and anxiety are highly prevalent among PWLWHIV lacking partner support (Tuthill et al., 2021; Waldron et al., 2022). A study in Tanzania found that 25% of such women met the criteria for depression, and 23.5% for anxiety (Ngocho et al., 2019). A lack of partner support for PWLWHIV can lead to increased risks of adverse birth outcomes, impaired infant development (Rodriguez et al., 2018b), nonadherence to ART (Lin et al., 2023), and higher neonatal mortality (Larsen et al., 2023), primarily due to the exacerbation of maternal depression, anxiety, and social isolation. Addressing these psychosocial factors is crucial for improving both maternal and neonatal health outcomes. A study in Tanzania found that antenatal depression was prevalent in 67% of the cohort, and was associated with a higher risk of infant wasting (relative risk [RR]: 2.61) (Saleh et al., 2023). Another study in Kenya highlighted that depressive symptoms were linked to a five-fold increase in pregnancy loss and a higher risk of preterm birth (Larsen et al., 2023).

The promotion of a positive birth experience has been a main goal of the World Health Organization (WHO)’s recent work on improving maternity care (WHO, n.d.). Improving maternal care to exceed maternal expectations leads to positive childbirth experiences and involves several key strategies grounded in evidence-based practices. First, ensuring respectful and dignified care is paramount (Mohamoud et al., 2023; Patel et al., 2024). Training healthcare providers in respectful maternity care (RMC) has been shown to significantly improve maternal satisfaction and birth experiences. This includes fostering a supportive environment where women feel valued and respected throughout their childbirth experience (Singh et al., 2024). Second, shared decision making and patient autonomy are critical. Many PWLWHIV feel excluded from decision-making processes regarding their care. They report that healthcare providers often do not seek their consent before procedures or involve them in care decisions, leading to feelings of disempowerment and a lack of autonomy (Alruwaili et al., 2024; Hughes et al., 2022; Jolly et al., 2019). Women value being active participants in their care, making informed decisions, and having their preferences respected. This can be facilitated through individualized care plans and effective communication from healthcare providers (Baumont et al., 2023; Downe et al., 2016). Third, continuity of care and emotional support are essential. Women benefit from consistent care providers and the presence of birth companions, which contribute to a sense of safety and support. Emotional support from both healthcare providers and companions can significantly enhance the childbirth experience (Downe et al., 2016; Yelland et al., 2009). Fourth, postnatal support is crucial for maternal well-being. Providing comprehensive postnatal care, including domiciliary visits and accessible support services, can address physical and emotional postpartum health needs (Hannon et al., 2022; Yelland et al., 2009). Lastly, addressing systemic healthcare issues such as healthcare provider training, resource availability, and infrastructure improvements can enhance the overall quality of maternal care. Ensuring adequate staffing, improving facilities, and implementing patient feedback mechanisms are important steps (Lazzerini et al., 2020; Mauluka et al., 2023; Valente et al., 2022). These strategies are supported by evidence from multiple studies and align with the World Health Organization’s standards for quality maternal care (Downe et al., 2016; Hannon et al., 2022; Lazzerini et al., 2020; Mauluka et al., 2023; Singh et al., 2024).

Structural and organizational issues such as overcrowding, lack of privacy, and inadequate facilities lead to negative childbirth experiences. PWLWHIV also report long waiting times and insufficient human resources, which can negatively impact the quality of care they receive (Lambert et al., 2018; Oyugi et al., 2024), which was also reported in our study.

Maternal mental health plays a significant role in the neurodevelopment of HIV-exposed uninfected (HEU) children. Maternal depression and stress are associated with adverse neonatal and neurodevelopmental outcomes in these children, mainly low birth weights and preterm birth (Grigoriadis et al., 2018; Portwood et al., 2023; Voit et al., 2022). Higher maternal depression scores are linked to lower overall cognitive scores in specific domains such as expressive language, fine motor skills, gross motor skills, and visual reception in HEU infants (Mebrahtu et al., 2018). Additionally, maternal stress is associated with poorer cognitive outcomes, particularly in expressive language, gross motor skills, and visual reception (Mebrahtu et al., 2018).

Maternal depression, both pre- and postpartum, has been shown to predict cognitive delays and gross motor development issues in HEU infants (Mokhele et al., 2019; Rodriguez et al., 2018a). Furthermore, maternal post-traumatic stress disorder (PTSD) and depression are significant risk factors for child behavior problems, with maternal PTSD having the greatest explanatory power for these issues (Nöthling et al., 2013). Persistent maternal mental health disorders, including depression and anxiety, are associated with lower cognitive and motor scores in infants (Burger et al., 2023). Suicidal ideation is also linked to poorer cognitive outcomes in children (Mebrahtu et al., 2020). Prohibiting having a birth companion during labor and delivery can contribute to feelings of isolation and anxiety (Hughes et al., 2022; Lambert et al., 2018).

Negative childbirth experiences can exacerbate postpartum mental health issues (Akinsolu et al., 2023; Larsen et al., 2023). Akinsolu et al. highlighted that having a partner was significantly associated with lower perceived depression among PWLWHIV as they provide emotional and psychological stability, reducing the risk of postpartum depression and mitigating the effects of previous negative childbirth experiences. This indicates that partner support can buffer the negative impact of adverse childbirth experiences on postpartum mental health (Akinsolu et al., 2023). A negative childbirth experience for PWLWHIV can also significantly influence their reproductive intentions and outcomes in subsequent pregnancies (Fortin-Hughes et al., 2019). 

This study had a limitation, which is that almost all PWLWHIV had a low viral load, which could be associated with better neonatal outcomes.

## 6. Conclusions

### 6.1. Practical Implications

PWLWHIV had positive childbirth experiences in public maternity wards in Rio de Janeiro. Our findings indicate that complications with baby health at birth was the main factor associated with a negative childbirth experience. Other studies have found that the perinatal period is a time of stress for all pregnant women, especially PWLWHIV (Hickson, 1998). The challenges experienced by PWLWHIV may compromise successful engagement in HIV care and may reduce the quality of life for these women and their children (Ashaba et al., 2017). The health of babies born to HIV-positive mothers is critically dependent on early HIV detection, effective ART, and careful management of the pregnancy and delivery to minimize MTCT and optimize neonatal outcomes.

Partner involvement is crucial for improving maternal and infant health outcomes in PWLWHIV through enhanced support, better ART adherence, and reduced MTCT rates; therefore, new approaches must be implemented involving PWLWHIV partners (Dinh et al., 2018; Sifunda et al., 2019).

### 6.2. Limitations of the Study

One of the limitations of our study was that we did not study postnatal child development. As we discussed above, prenatal through to postnatal care is important in child development. Another limitation was not assessing maternal mental health using specific scores, such as the Edinburgh Postnatal Depression Scale, a 10-item self-rated scale (Kroska & Stowe, 2020; Miller et al., 2023).

Another limitation is the small sample used, but it is representative of the population of Rio de Janeiro, which was ensured by the sample calculation.

### 6.3. Recommendations for Future Research

Future research on improving childbirth experiences for PWLWHIV should include approaches targeting emotional, social, and health factors. Maternity service providers should consider a multi-faceted approach to reorient pre- and postnatal services in order to improve PWLWHIV’s experiences of care. Approaches worthy of consideration include ensuring consistency and continuity of care through staffing arrangements; guidelines and protocols based on PWLWHIV’s decision making; an emphasis on planning for postnatal care during pregnancy; the use of evidence to inform both consumer information and advice, and in the practice of providing care; and skill-enhancement opportunities for improving healthcare professional communication with PWLWHIV.

Interventions targeting reductions in depressive symptoms in perinatal HIV-positive women could include increasing partner involvement and decreasing HIV stigma and intimate partner violence (Peltzer et al., 2020).

Addressing antenatal depression and anxiety through cognitive behavioral therapy (CBT) and other psychosocial interventions could also significantly reduce depressive symptoms, which are associated with adverse birth outcomes (Jacobson et al., 2021; Mendelson et al., 2017; Mphonda et al., 2023; Sakyi et al., 2020).

### 6.4. Ethical Considerations

Human ethics approval was granted from the IPPMG and CONEP (National Commission in Research Ethics). All the PWLWHIV who enrolled in the study met the inclusion criteria, and this study was explained to them. They were informed that their participation was voluntary, and their responses were confidential. Written informed consent was then obtained, and data collection was completed. Before beginning the interviews, we ensured that the women understood the information provided, consented to participate, and allowed the extraction of their data for the specified aims. The data were pseudo-anonymized before the analysis. This study complied with the guidelines for human research in the Declaration of Helsinki.

## Figures and Tables

**Table 1:** Sociodemographic characteristics of the sample (N = 82).

Variable	Value
Mother	
Maternal age at birth	28.5 [23.3; 28.6], 19–49
Ethnicity	
White	14 (17.1%)
Mixed	34 (41.5%)
Black	34 (41.5%)
Number of pregnancies	1.5 [1; 3], 1–6
Number of previous deliveries	0 [0; 2], 0–7
Planned pregnancy	
No	69 (84.1%)
Yes	13 (15.9%)
Viral load at 34 weeks	
Detectable	1 (1.2%)
Undetectable	79 (96.3%)
Undetermined	2 (2.4%)
Birth type	
Cesarean	25 (30.5%)
Vaginal	57 (69.5%)
Baby	
Age (weeks)	
38	20 (24.4%)
39	32 (39.0%)
40	24 (29.3%)
41	6 (7.3%)
Sex	
Female	40 (48.8%)
Male	42 (51.2%)
Weight	3180 (247), 2580–3820
Apgar score at 1 min	9 [8; 9], 1–9
Apgar score at 5 min	9 [9; 9], 2–10
Birth complications	
No	75 (91.5%)
Yes	7 (8.5%)

Results are described using absolute and relative frequencies (n (%)); median [1st quartile; 3rd quartile]; and mean (SD), min–max.

**Table 2:** Reasons given by PWLWHIV to explain their childbirth experience (N = 82).

Group	Answers	n (%)
Positive reasons		
Baby health	Baby was crying	33 (40.2)
	Healthy baby	
	Baby’s well-being	
	Baby’s birth	
	Saw the baby’s face	
	Had skin-to-skin contact with baby	
Companion presence	Husband was present	26 (31.7)
	Mother was present	
	Husband could not enter delivery room	
	Happiness of husband	
	Support from husband	
	Absence of a partner	
Good healthcare professional support	Support from nurses Wonderful doctors	25 (30.5)
	Excellent doctors	
	Excellent nurses	
	Attention from doctors	
	Good support from doctors	
	Attention from healthcare professionals	
	Wonderful nurses	
	Given respect from all healthcare professionals	
	Excellent anesthetist	
Good physical structure of maternity ward	Good hygiene in maternity ward	8 (9.8)
	Good structure of maternity ward	
Everything went well	Everything went well	4 (4.9)
	Everything was perfect	
	Fully assisted in maternity ward	
Did not feel pain	Did not feel pain during birth	1 (1.2)
Prenatal visit to maternity ward	Visited maternity ward before the childbirth	1 (1.2)
Negative reasons		
Poor healthcare professional support	Poor care from doctors	12 (14.6)
	Poor care from nurses	
	Poor care from nurses and doctors	
	Lack of attention from nurses	
	Absence of nurses during labor	
Companion absence	Absence of mother	6 (7.3)
	No companion	
	Absence of husband	
Excessive pain or medication	Terrible expulsive period	7 (8.5)
	Large amount of bleeding and pain	
	Painful birth	
	Long fast	
	Excessive pain	
Poor physical structure of maternity ward	Bed was not secure	1 (1.2)
Absence of birth information	Absence of birth information	1 (1.2)
Complications for baby	Baby was suffering	3 (3.7)
	Baby went to UTI	
	No contact with baby	
Cesarean delay	Cesarean delay	2 (2.4)

## Data Availability

Further inquiries about original data can be asked directed to the corresponding author.

## Supplementary Materials

The following supporting information can be downloaded at: https://www.mdpi.com/article/10.3390/bs15040442/s1, Table S1: Frequency of positive and negative experience of the sample (N = 82). Table S2. Frequency of PWLWHIV reasons to classify their childbirth experience (N = 82). Figure S1. Frequency of positive experience, regarding if it is the first baby or not.
