# Supplementary material for: Major Factors Contributing to Positive and Negative Childbirth Experiences in Pregnant Women Living with HIV
Source: Behav Sci (Basel). 2025 Mar 31;15(4):442. doi: 10.3390/bs15040442 (PMC12024450; doi:10.3390/bs15040442)

Supplementary tables

Table S1. Frequency of positive and negative experience of the sample (N=82).

	negative	neither positive nor negative	positive
**Childbirth experience**	14 (17.1%)	3 (3.6%)	65 (79.3%)

Table S2. Frequency of PWLWHIV reasons to classify their childbirth experience (N=82).

**Groups**	**n(%)**
**Positive reasons**	
Baby health	33 (40.2)
Companion presence	26 (31.7)
Good health care professional support	25 (30.5)
all things went well	4 (4.9)
perfect physical structure of maternity	8 (9,8)
not feeling pain	1 (1,2)
Early visit maternity	1 (1,2)
**Negative reasons**	
Bad health care professional support	12 (14.6)
Companion ausence	6 (7.3)
Excessive pain or medicalization	7 (8.5)
Bad physical structure of maternity	1 (1.2)
Absence of birth information	1 (1.2)
Complication with baby	3 (3.7)
Cesarean delay	2 (2.4)

Figure S1 . Frequency of positive experience, regarding if it is the first baby or not

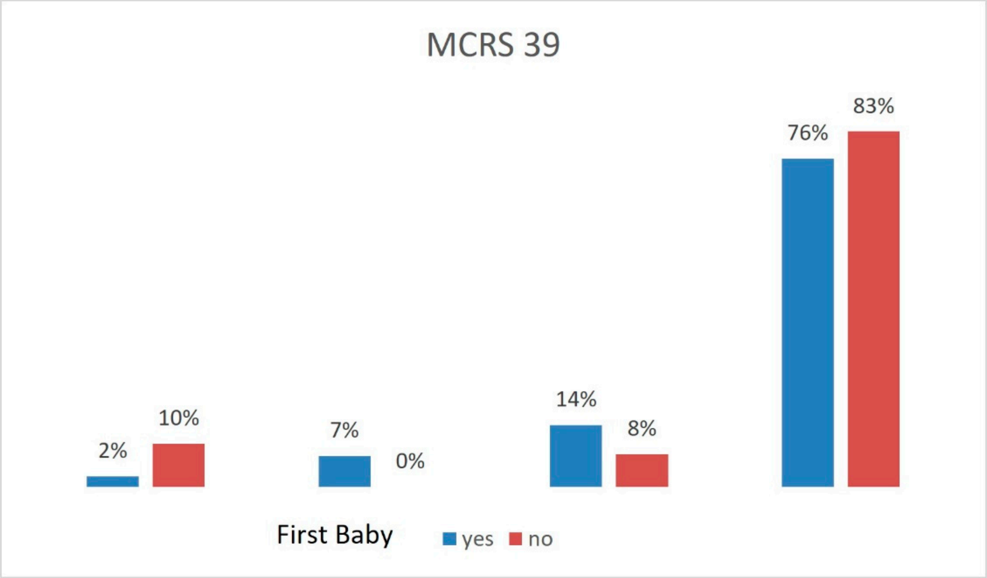

Supplement: Supplementary file 1 [file behavsci-15-00442-s001.zip › behavsci-3500606-supplementary.pdf]
